# Role of the Molecular Tumor Board for the Personalized Treatment of Patients with Metastatic Breast Cancer: A Focus on the State of the Art in Italy

**DOI:** 10.3390/cancers15061727

**Published:** 2023-03-12

**Authors:** Azzurra Irelli, Sofia Chiatamone Ranieri, Daniela Di Giacomo, Sara Malatesta, Leonardo Valerio Patruno, Alessandra Tessitore, Edoardo Alesse, Katia Cannita

**Affiliations:** 1 Medical Oncology Unit, Department of Oncology, AUSL 04 Teramo, 64100 Teramo, Italy; 2 Pathology Unit, Department of Services, AUSL 04 Teramo, 64100 Teramo, Italy; 3 Department of Biotechnological and Applied Clinical Sciences, University of L’Aquila, Via Vetoio, 67100 L’Aquila, Italy

**Keywords:** precision oncology, molecular tumor board, next-generation sequencing, metastatic breast cancer, genomic alterations, agnostic anticancer drugs

## Abstract

**Simple Summary:**

Molecular tumor boards (MTBs) aim to translate the results of genomic tests into clinical practice in order to set-up the most appropriate therapeutic strategy for the individual patient. Genomic tests represent a challenge for the Italian National Health System, which must set-up new paths for access to medicines and, at the same time, guarantee economic sustainability. Furthermore, the scientific and clinical skills of clinicians must be guaranteed to follow the complex and rapid evolution of cancer genetics and the availability of agnostic anticancer drugs. This review aims to analyze the role of MTBs in the management of patients with metastatic breast cancer (MBC).

**Abstract:**

Molecular tumor boards (MTBs) are multidisciplinary groups that combine molecular and clinical data from cancer patients in order to formulate treatment recommendations for precision medicine. To date, there is insufficient data to support the use of singleplex or next-generation sequencing (NGS) technologies to select first-line therapy for patients with metastatic breast cancer (MBC), but considering the high number of level II alterations, according to the ESMO scale for clinical actionability of molecular targets (ESCAT), it is suggested to include patients in molecular screening programs in order to be able to offer targeted therapies for specific genomic alterations. This article aims at reviewing the most recent literature related to the most used methodologies/approaches for molecular diagnostics and variants’ classification, summarizing the internationally published molecular screening studies in support of MTB activity and, in the end, discussing MTBs’ current position and role in Italy, the number of which is increasing, also thanks to the thrust of institutions.

## 1. Introduction

Breast cancer is the most diagnosed malignancy in women. In 2020, 2.3 million new cases of breast cancer were estimated, and there were 683,000 deaths from this disease, while approximately 7.8 million women were alive with a diagnosis of breast cancer [1]. In Italy, in 2020, approximately 55,000 new diagnoses of breast cancer and approximately 12,300 deaths were estimated; in addition, more than 834,000 women diagnosed with breast cancer were alive.

The incidence of breast cancer increases with age. In particular, the incidence increases until menopause and then slows down after, and starts to rise again after the age of 60 [2,3]. The risk factors include age, reproductive factors, hormonal factors, dietary and metabolic factors, previous thoracic radiation therapy, previous dysplasia or breast cancer, family history, and genetic factors [1].

The choice of systemic therapy in patients with metastatic breast cancer (MBC) depends on several factors, such as the biomolecular profile of the tumor, disease burden, symptoms, ECOG performance status, comorbidities, socioeconomic status, and patient preferences [4].

MBC still remains incurable despite continuous and gradual improvement in progression-free survival (PFS) and/or overall survival (OS) achieved with different targeted therapies. The biomolecular stratification of breast cancer is based on the immunohistochemical staining of estrogen receptors, progesterone receptors, human epidermal growth factor receptor 2 (HER2), proliferation marker Ki-67, and, in the case of triple-negative breast cancer, of the programmed death ligand (PDL1). Tumor DNA sequencing in MBC is currently not yet common in clinical practice and is mostly reserved for the determination of the mutational status of the *BRCA1/BRCA2*, *PIK3CA*, and, more rarely, the *ESR1* and *ERBB2* genes [5,6].

Next-generation sequencing (NGS) has provided new opportunities for precision oncology. The interpretation of NGS data and their application in clinical practice is entrusted to multidisciplinary groups called molecular tumor boards (MTBs), as experimented for the first time by Michigan University. The purpose of MTBs is to identify potential therapeutic strategies based not only on genetic analysis but also on the patient’s clinical characteristics (age, performance status, and comorbidity) [7].

It is also necessary to know the outcomes of the patients discussed in the MTBs who never reach the treatment stage in order to improve the processes and, therefore, the opportunities for the patients [8].

Despite the availability of NGS technology and targeted therapies, the use of precision medicine remains scarce due to the insufficient support to guide clinicians in managing data from NGS. Many centers have instituted MTBs, although data to support their clinical utility are currently weak [9].

**Figure 1:**
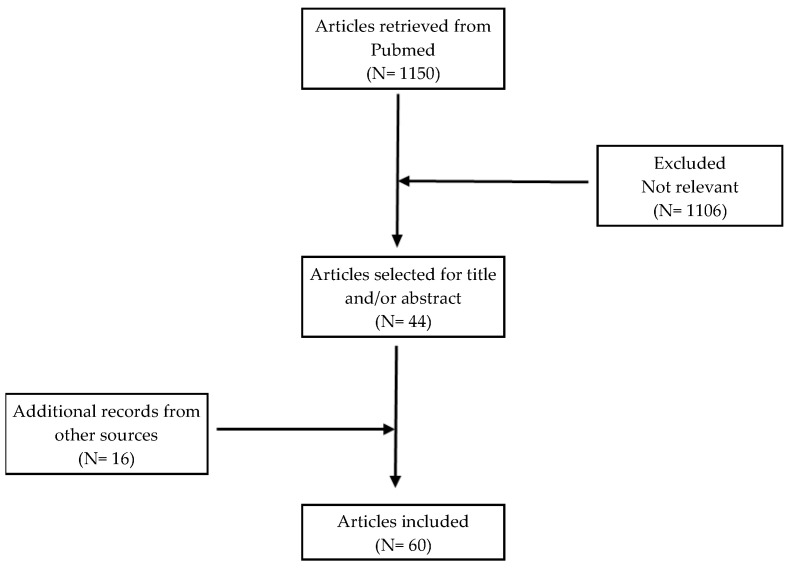
Flow chart of the methodology.

A PubMed search was conducted on 4 March 2023 using the query “Molecular Tumor Board AND breast cancer”. Further articles were identified by reading the bibliography of the selected articles. The aim of this review was to provide an analysis of the role of MTBs in the management of patients with MBC (Figure 1).

## 2. New Strategies for Precision Oncology

Precision oncology has seen the succession of three different therapeutic approach strategy models: histological, agnostic, and mutational.

In the histological model, the starting point is represented by the organ from which the disease originates, with the histological examination and the identification of protein alterations through traditional immunohistochemical techniques. Such a classical approach is now supported and integrated by the mutational model based on of the use of molecular technologies (e.g., next-generation sequencing (NGS)) for the identification of well-determined biomarkers aimed at adopting drugs matching specific gene lesions.

The agnostic approach, applied to any type of cancer characterized by a specific mutational landscape predicting the response to specific treatments, regardless of its primary site, fits into this context.

Furthermore, the mutational model also includes the possibility of studying alterations of the microbiota (i.e., the set of billions of billions of microorganisms that live in humans) by considering the presence of any concomitant pathologies and their treatments [10].

Molecular diagnostic technologies, aimed at identifying genomic alterations, are divided overall into “singleplex”, such as the real-time PCR methods, which analyze known variants in well-defined target genes, and next-generation sequencing (NGS), a very powerful methodology that not only allows the simultaneous analysis of panels of genes, even hundreds, providing a high depth of coverage, but also of whole exomes or genomes (e.g., the FoundationOne^®^ (CDx) test, more than 300 genes at the same time; Caris Molecular IntelligenceTM). Both real-time PCR and NGS show high sensitivity and a low limit of detection (LOD), making it possible to determine the presence of variants at low frequency (below 5%), thus uncovering the intra-tumor heterogeneity [11,12,13,14].

NGS makes possible the identification of both known and unknown variants, driver, passenger, actionable, and druggable mutations, as well as the tumor mutational burden (TMB). Moreover, the NGS analysis of mRNA/cDNA can also be applied to the study of rearrangements/deletions/gene fusions in several targets with clinical/diagnostic relevance (e.g., *ALK*, *ROS1*, *RET*, *NTRK1/2/3*, *FGFR2/3*, *PPARG*, and *MET*).

In this context, bioinformatics data processing using specific pipelines is essential to obtain high-quality results [13].

For molecular assays, DNA from an FFPE tumor tissue is preferred due to the fact of its easier availability and greater abundance; however, over the last few years, several diagnostic approaches have been based also on the analysis of the “so-called” liquid biopsy, consisting of tumor-derived entities, in particular circulating tumor DNA (ctDNA, cfDNA), poured into the bloodstream. Therefore, the analysis of cfDNA is minimally invasive and can also be considered an alternative when cancer tissue is unavailable/inadequate for molecular procedures or the patient has comorbidities that contraindicate an invasive diagnostic approach. Moreover, at relapse and in patients undergoing treatment with target drugs, liquid biopsy shows the advantage of better representing tissue heterogeneity, highlighting the onset of mechanisms of acquired therapy resistance in cases where there is a need for clinical application. However, when interpreting liquid biopsy test results, the possibility of false negatives (e.g., no spread of tumors) or false positives (e.g., sequence artifacts) should be considered. Furthermore, the detection of complex gene alterations by liquid biopsy poses further problems concerning the diagnostic sensitivity (i.e., fusions) and accuracy (i.e., copy number variant).

There are no clear international recommendations on the most appropriate tissue (primary vs. metastasis) or the best timing (at diagnosis vs. disease progression) for performing NGS testing. Experts believe that, if available, NGS testing should be performed on the most recent available tumor specimen.

An NGS analysis for biomarkers used in clinical practice could reveal the presence of germline genetic alterations predisposing to cancer as well. It is important to underline that the mutations identified in the tumor tissue cannot be considered of germline origin in the absence of contextual confirmation by a test carried out on nontumor tissue, usually peripheral blood leukocytes. Given the important implications of germline mutations, not only in affected patients but also in unaffected relatives in terms of increased cancer risk, patients framed for carrying a suspected germline mutation should be referred for genetic counselling.

A biological, pathological, and clinical interpretation of the identified variants is also necessary, with particular attention to variants of uncertain significance, whose classification needs re-evaluation over time [15].

## 3. Variant Classification Scales

In 2014, Andre et al. developed a scale to rank genomic alterations according to the likelihood of being therapeutic targets. In this scale, the level of evidence (LOE) combines two levels of evaluation: the first defines the robustness of the data source (from level I, which requires that the molecular alteration is validated by at least one randomized phase III study, to level IV which states that clinical and preclinical data are lacking for the validation of the molecular alteration); the second defines the relevance of the information with respect to the disease considered, from the most relevant (A) to the least relevant (C) [16].

Another scale was described, in 2014, by Van Allen et al. comprising five LOEs (from A to E) informing whether the genomic alteration has a validated association, limited clinical evidence for the type of tumor considered, limited clinical evidence for another type of tumor, limited evidence for preclinical, or an inferential association; this in relation to four possible roles: predictive for FDA-approved therapies, predictive from clinical trials, prognostic, and diagnostic [17].

A different classification example was presented, in 2015, by Dienstmann et al., who devised a “clinical targetability index”, with the consequent evaluation of genomic alterations at multiple levels (gene, variant, type of tumor, and type of drug) [18].

In 2016, Sukha et al. proposed a classification of somatic variants in metastatic tumors, organized into five categories based on known or expected pathogenicity of the variant, primary site, and histology of the tumor to which the variant belongs, variant recurrence, and evidence of its clinical feasibility [19].

In 2017, Chakravarty et al. created “OncoKB”, a database of somatic mutations approved by the Food and Drug Administration (FDA) based on their prognostic and predictive significance [20].

The joint consensus (JCR) of the Association for Molecular Pathology (AMP), the American College of Medical Genetics and Genomics (ACMG), the American Society of Clinical Oncology (ASCO), and the College of American Pathologists (CAP) for reporting genetic variants in cancer is an additional scale of the classification of molecular targets into levels of potential clinical utility [21].

**Figure 2:**
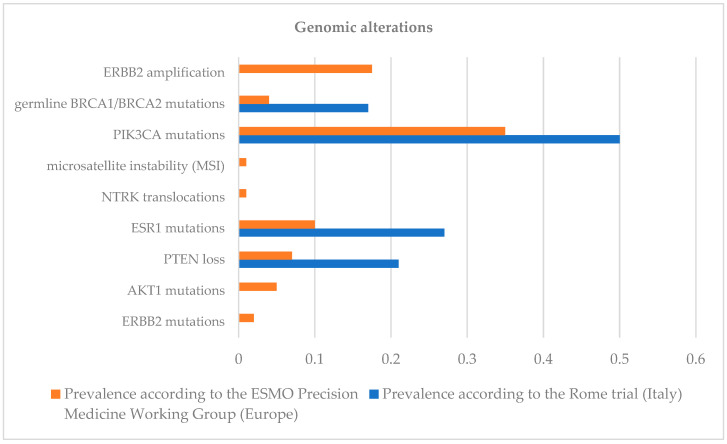
Prevalence of level I/II genomic alterations of metastatic breast cancer according to the ESCAT scale in European [23] and Italian [24] populations.

The ESMO scale for clinical actionability of molecular targets (ESCAT) assigns each genomic alteration at a level of clinical utility based on its potential use as a therapeutic target. The ESCAT scale has six distinct levels (I–V and X) based on the strength of evidence from clinical trials. The first two levels are of the greatest clinical relevance: level I includes alterations for drugs ready for use in clinical practice, while the alterations for which there are drugs capable of conferring a clinical benefit, according to subgroup analyses of clinical studies, belong to level II. According to the ESCAT scale, the level I genomic alterations of MBC include *ERBB2* amplification, germline *BRCA1/BRCA2* mutations, *PIK3CA* mutations, microsatellite instability (MSI), and *NTRK* translocations. Level II genomic alterations include *ESR1* mutations, *PTEN* loss, *AKT1* mutations, and *ERBB2* mutations (Table 1) (Figure 2) [22,23,24].

The abovementioned reference scales assist the MTB in recommending the most appropriate therapeutic treatment [7].

## 4. New Clinical Trial Designs

Two new clinical trial designs were developed to accelerate the development of agnostic drugs: the umbrella study and the basket study.

In the umbrella study, patients with cancer of single-organ origin are assigned to different treatment arms, based on the identified genomic alteration, and treated with drugs targeting specific genomic alterations. The umbrella study includes patients with a single histology and then distributes them based on multiple biomarkers. The strength of this approach makes it possible that, when the prevalence of biomarkers is low, the screening success rate improves with more arms, and the flexible protocol design allows investigators to easily add into the arm. On the other hand, the large number of drugs and biomarkers can show a challenge.

Conversely, in the basket study, patients are recruited based on molecular characteristics, thus even tumors originating from different organs can receive the same treatment. The basket study, therefore, uses a single treatment and a single biomarker across different histology types and anatomical sites. The basket study is typically based on the recruitment of few patients, and there is no control group [25,26].

## 5. Molecular Screening Studies

The MOSCATO-01 study is a single-center French cancer screening program on biopsies from metastasis sites in patients with various metastatic malignancies including breast cancer (135 out of 948 enrolled patients, 14%). Four hundred and eleven out of eight hundred and forty-three evaluable patients (49%) showed a target molecular abnormality, but only one hundred and ninety-nine patients (24%) received targeted therapy according to MTB indications. Other patients did not receive targeted therapy due to the fact of rapid clinical deterioration (64 patients), inclusion in another study (45 patients), and exclusion criteria (21 patients). The primary endpoint of the study was met, with PFS2 (progression-free survival on therapy decided by MTB)/PFS1 (progression-free survival on therapy before that decided by MTB) > 1.3. Furthermore, 63 out of 948 patients (approximately 7%) benefited from the target drug in PFS [27].

In the Safir01/UNICANCER study, a French multicenter molecular screening study in MBC, genomic alterations in individual patients were identified with the aim of providing targeted therapy matched to genomic alterations. The primary endpoint was to include 30% of patients in clinical trials testing targeted therapy and, therefore, the primary outcome was the proportion of patients who could be offered targeted therapy. Four hundred and twenty-three patients were included, and evaluable biopsy specimens were identified for 407 patients. Readable genomic tests were available for 297 patients. A target genomic alteration was identified in 195 (46%) patients, most frequently *PIK3CA* (74 (25%) of 297), *CCND1* (53 (19%)), and *FGFR1* (36 (13%)). One hundred and seventeen (39%) of two hundred and ninety-seven patients had rare genomic alterations (defined as present in less than 5% of the general population), including *AKT1*, *EGFR*, *MDM2*, *FGFR2*, *AKT2,* and *IGF1R* mutations, and *MET* amplifications. Therapy was personalized in 55 (13%) of 423 patients. Among 43 patients who were evaluable and received targeted therapy, four (9%) had an objective response and nine (21%) had stable disease for more than 16 weeks. The lack of access to targeted therapy for patients may lead to this lower percentage [28].

Four hundred and eighty-three patients with MBC were prospectively enrolled at the Princess Margaret Cancer Center in Canada, and 440 of these were genotyped. At least one somatic mutation was identified in 46% of patients, most commonly in *PIK3CA* (28%) or *TP53* (13%). Among 203 patients with at least one mutation, 15% were treated in genotype-matching studies and 9% in nonmatching studies. There was no significant difference in the median time on treatment among patients treated with matched therapies and those with unmatched therapies (3.6 vs. 3.8 months; *p* = 0.89) [29].

The SOLTI-1301 AGATA study aimed to evaluate the feasibility of a multicenter molecular screening program in Spain to better characterize the genomic landscape of advanced breast cancer and facilitate patient access to matched targeted therapies. Three hundred and five patients with advanced breast cancer from 10 institutions were enrolled. Tumor sequencing was successful in 260 (85.3%) patients. Somatic mutations were found in 163 (63%) patients; 76% of them were potentially targetable. Somatic mutations were identified mainly in the *PIK3CA* (34%) and *TP53* (22%) genes. Moreover, *AKT1* mutations were more prevalent in metastatic samples than in primary tumor samples (9% vs. 2%; *p* = 0.01). Genome-guided cancer therapy was recommended in 45% (n = 116) of patients screened, 11% (n = 13) of whom ultimately received it. Among these patients, 46.2% had PFS ≥ 6 months with combination therapy. The reasons for not receiving the recommended targeted therapy were related to the physician’s or patient’s decision, clinical trial screening failure, or rapid disease progression/death. Furthermore, data suggest that it is preferable to match patients with potential therapies based on an analysis of their metastatic tumor rather than their primary tumor, because metastatic tumors often develop new mutations during the metastasis process and treatment reinforcing the need of biopsies from metastatic sites [30].

Forty-three patients with heavily pretreated advanced breast cancer were studied by the University of California MTB, UC San Diego Moores Cancer Center. Forty out of forty-three patients (93%) had at least one theoretically feasible aberration (mean, 4.79 abnormalities/patient). Seventeen out of forty-three patients (40%) were treated accordingly to MTB decision: seven (16% of 43, or 41% of 17) achieved stable disease (n = 2) or partial remission (n = 5) for 6 or more months. Most of the patients were not treated according to the indications of the MTB, mainly because their tumors had not yet progressed with the previous therapy or because of death, unavailability of drugs, and choice of the physician. An analysis of the relationship between PFS2 and PFS1 showed no difference for patients treated with the indicated therapy, but PFS was significantly worse for patients receiving a therapy not matched with the identified genomic alteration [31].

In a retrospective analysis by Walter et al., 52 patients with advanced breast cancer were candidates for tumor tissue sequencing. Each sample showed at least one affected gene, with targetable mutations in 45 out of 52 patients (87%). New treatment options were found in 22 out of 52 patients (42%). The relevant pathogenic germline variants were also detected in 9.6% (5/52) of patients. The reported genes mainly belong to the PI3K/AKT/mTOR and FGFR/ERBB/EGFR pathways [32].

The Breast International Group (BIG) is conducting AURORA (AURORA: Aiming to Understand the Molecular Aberrations in Metastatic Breast Cancer), a molecular screening program that aims at improving the understanding of MBC through broad profiling of primary tumors matched to metastatic samples, as well as circulating free DNA (cfDNA) extracted from plasma, collected from at least 1000 patients. The design of AURORA is based on the prospective collection of matched primary and metastatic specimens in patients who are treatment naive for MBC or after one line of therapy. From the analysis of the first 381 patients, most of the driver mutations were shared (88%), and only a minority of patients (10%) had at least one exclusive mutation in the metastatic sample. This is in contrast to other studies that reported a higher rate of acquired genomic alterations, ranging from 45% to 73%. It has recently been reported that mutations in metastases are less associated with metastatic spread and accumulate under the selective pressure of cancer therapies. While a higher TMB in MBC has been associated with worse outcome, AURORA is the first study to show that high TMB is associated with shorter time to progression after HR+/HER2- early breast cancer treatment. Despite the fact that at least 51% of patients had an ESCAT level I or II abnormality (83% of patients if we include level III and IV abnormalities), only 7% of patients in this cohort received therapy combined with molecular data, probably due to the lack of availability of targeted drugs. The results from the cfDNA analysis in AURORA broadened the panorama of targetable mutations (11% of cases with unidentified alterations in a tissue sample). However, targetable changes may not be detected in plasma (40% changes in AURORA) due to the lower variant allelic frequency (VAF) in tissue samples [33].

The phase 2 SHIVA study enrolled 741 patients with any type of metastatic solid tumor progressing from standard therapy for whom a molecular alteration was identified within three molecular pathways (hormone receptors, PI3K/AKT/mTOR, and RAF/MEK) and, therefore, eligible for one of the following ten drugs: erlotinib, lapatinib plus trastuzumab, sorafenib, imatinib, dasatinib, vemurafenib, everolimus, abiraterone, letrozole, and tamoxifen. One hundred and ninety-five of two hundred and ninety-three evaluable patients (20% with breast cancer) were randomized to receive a targeted agent (experimental group, 99) or an agent of physician’s choice (control group, 96). At a median follow-up of 11.3 months, PFS (primary endpoint) was 2.3 months in the experimental group versus 2.0 months in the control group (HR = 0.88; *p* = 0.41). Thus, the use of targeted drugs did not statistically significantly improve PFS. Despite the small number of targeted therapies available and negative results, 40% of included patients received the molecular matching therapy [34].

In a pooled population analysis of the phase II studies SAFIR02-BREAST and SAFIR-PI3K, patients with HER2-negative MBC who were candidates for first- or second-line chemotherapy (thus resistant to hormone therapy if hormone receptor positive) underwent a biopsy of a metastatic site, and genomic analysis of that tissue was performed. After 6–8 cycles of chemotherapy, patients with no disease progression and a target genomic alteration were randomized to targeted therapy versus maintenance chemotherapy. Among 1462 evaluable patients, 238 (16%) were subsequently randomized between maintenance chemotherapy (n = 81) and targeted therapy (n = 157). In the 115 patients with the genomic abnormality ESCAT I/II, median PFS was 9.1 and 2.8 months in the targeted therapy and chemotherapy arms, respectively (HR = 0.41; *p* < 0.001). In the overall population (n = 238), there was no statistically significant difference in PFS between the two treatment arms (5.5 vs. 2.9 months; HR = 0.77; *p* = 0.109). Thus, the use of multigene sequencing as a therapeutic decision-making tool improves outcomes for patients with MBC only in the case of alterations classified as ESCAT I/II [35].

CATCH (comprehensive assessment of clinical features and biomarkers to identify patients with advanced or metastatic breast cancer for marker driven trials in humans) is a prospective single-center study using genomics and transcriptomics to guide treatment decisions in the clinical management of MBC. The genomic alterations affecting the biomarkers were single nucleotide variants, insertions and/or deletions, homozygous amplifications, and deletions with a variation of the copy number and expression aberrations. The biomarkers analyzed were stratified according to the cellular pathways they belong to or the class of drugs they are aimed at (PI3K-AKT-mTOR, nuclear receptors, tyrosine kinases, immune checkpoint inhibition, MAPK pathway, cell cycle, and DNA damage response and ADC targets). The treatments implemented according to the MTB indication saw the use of drugs such as PI3K-AKT-mTOR inhibitors, endocrine therapy, pan-TKI, DNA-damage response drugs, ICI, CDK4/6 inhibitors, and anti-HER2/Pan-ERBB inhibitors. Among the 200 enrolled patients, 128 (64%) were discussed in MTB of whom 64 (50%) were subsequently treated according to the MTB recommendation. Among 53 evaluable patients, 21 (40%) achieved stable disease (n = 13.25%) or partial response (n = 8.15%) with the MTB’s chosen target drug. Furthermore, 16 (30%) of those patients showed PFS improvement of at least 30% during MTB-recommended treatment compared to PFS of the previous line of treatment, demonstrating that precision oncology provides clinical benefits [36].

The retrospective study by Bruzas et al. included 95 patients with MBC. Genomic alterations were identified in all tumor samples, and 83 patients (87.4%) had a median of two (range, 1–6) potentially targetable alterations. The MTB recommended genomic-guided therapy to 63 patients, 30 of whom received such treatment. The ratio of PFS in NGS-based therapy to PFS in the last line of standard therapy before NGS was >1.3 of 13 (43.3%) patients, indicative of a clinical benefit to NGS-directed therapy. The one-year overall survival rates were 22.7% in the 65 patients assigned to standard therapy compared with 62.9% in the 30 patients who received combination therapy. Thus, treatment combined with NGS improved clinical outcomes in a subset of patients with MBC [37].

Among 310 patients who underwent molecular analysis in a Japanese hospital by the group of Fukada et al., 35 patients with MBC were studied after excluding two cases that could not be analyzed. Targetable gene mutations were detected in 30 patients (85.7%), and 7 patients (20.0%) were recommended for participation in the clinical trial, with the drug administered to 2 patients (5.7%). Three patients (8.6%) died due to the fact of disease progression prior to the release of the test results [38].

In the open-label, multicohort, phase 2a plasmaMATCH study conducted in 18 UK hospitals, patients had completed at least one prior line of treatment for advanced or relapsed breast cancer within 12 months of neoadjuvant or adjuvant chemotherapy. Patients were recruited into four parallel treatment cohorts matched to identified ctDNA mutations: cohort A included patients with *ESR1* mutations (treated with fulvestrant); cohort B included patients with *HER2* mutations (treated with neratinib and, if estrogen receptor positive, with fulvestrant); cohort C included patients with *AKT1* mutations and estrogen receptor positive cancer (treated with capivasertib plus fulvestrant); and cohort D included patients with *AKT1* mutations and estrogen receptor-negative cancer or *PTEN* mutation (treated with capivasertib). One thousand fifty-one patients were enrolled for the study, with ctDNA results available for one thousand thirty-four patients. The agreement between digital PCR of ctDNA and targeted sequencing was 96–99%. Cohorts B and C met or exceeded their target number of responses, with five (25%) of 20 patients in cohort B and four (22%) of 18 patients in cohort C having a response. Cohorts A and D fell short of their target number of responses, with 6 (8%) of 74 in cohort A and two (11%) of 19 patients in cohort D having a response. The results demonstrate the clinically relevant activity of targeted therapies against rare *HER2* and *AKT1* mutations, confirming that these mutations could be targets of oncological treatments for MBC [39].

The group of van Geelen et al. recruited patients with advanced breast cancer of any subtype to perform NGS analysis on the most recent tumor samples, using a panel of 108 breast cancer-specific genes. Three hundred and twenty-two patients were enrolled in the study, with 72% (n = 234) of patients successfully sequenced (n = 357). The majority (74%, n = 171) of patients sequenced were found to carry a potentially targetable alteration, the most common of which is a *PIK3CA* mutation. Alterations were found in *AKT1*, *BRCA2*, *CHEK2*, *ESR1*, *FGFR1*, *KMT2C*, *NCOR1*, *PIK3CA*, and *TSC2*. Seventy-four (43%) patients with targetable changes were referred to a clinical trial or had germline confirmatory testing or had a change in therapy outside of clinical trials. Agreement between primary and metastatic samples for key driver genes (*TP53*, *ERBB2* amplification) was >75%. It was observed that patients with a higher number of mutations had significantly worse overall survival [40].

The Rome Trial is an example of a randomized phase II basket study underway in Italy which involves the enrollment of 1200 patients with different types of metastatic cancer progressing from two (or three, only for some types of cancer) standard lines of treatment. This study involves the use of Roche’s Navify platform for data collection and interdisciplinary discussion within the MTB [41]. The objective of the study is to evaluate the efficacy and safety of a target treatment versus standard of care in patients with solid tumors. Preliminary results in the MBC cohort were reported at SABCS 2022. Patients with MBC who received at least one and no more than two systemic treatments were enrolled. Mutational data are available for sixty-two patients in the MBC cohort. NGS was available on both tissue and liquid biopsy in 48 (77%) patients, and 14 had only liquid biopsy available due to tissue test failure. Some pathways were frequently altered: PIK3CA/AKT/MTOR (60%), TP53 (60%), Cell cycle/cyclin (35%), FGF/FGFR (26%), BRCA1/BRCA2 (17%). The most frequently altered genes were: *TP53* (61%), *PIK3CA* (50%), *ESR1* (27%), *CCND1* (27%), *FGF19* (24%), *FGF3* (24%), *FGF4* (22%), *MYC* (22%), *FGFR1* (21%), *PTEN* (21%), *EMSY* (16%), *RB1* (14%), *RAD21* (14%), *TET2* (13%), *BRCA2* (11%), *GATA3* (11%), and *KRAS* (10%). No patients with MSI status were reported. Eight (13%) had high TMB (>10). Actionable mutations were detected in 34 patients (54%). Twenty-eight (45%) patients were assigned to a target drug after MTB discussion: ipatasertib (16), pemigatinib (5), ipilimumab plus nivolumab (4), lapatinib plus trastuzumab, TDM1, and everolimus (1). MTB required a germline test for six patients; four were confirmed (66%; two *BRCA*, one *PALB2*, and one *BRIP1*). Approximately 15% of patients had elevated TMB, but MSI is confirmed as a rare event in breast cancer. Germline mutations have been identified in patients with no prior indication for germline testing [24].

Thus, the genomic profile of patients with MBC may have clinical implications and should be considered in all eligible patients (Table 2).

## 6. MTBs: Current Status and Future Prospects

MTBs are multidisciplinary groups that examine the molecular and clinical data of patients in order to formulate therapeutic recommendations [42] based on the available scientific evidence [43].

MTBs were designed with the goal of supporting clinical decision making.

MTBs are typically composed of a multidisciplinary team, including medical oncologists, surgical oncologists, geneticists, pathologists, pharmacists, radiologists, and other basic scientists, who collaborate to provide patient recommendations for targeted therapies, such as the opportunity to participate in clinical trials. Geneticists then allow MTBs to provide recommendations for genetic testing in cases where a potential pathogenic germline mutation is identified, with consequent possible repercussions on the family members of patients, eligible for early cancer screening [44].

From a literature review examining a total of 51 articles from 11 different countries over a 6-year period, 20% of patients received targeted treatment with MTB [8].

Patients who received oncological therapy based on the MTB decision, when a targetable genomic alteration is identified, had better outcomes such as overall survival, as described in a cohort from the San Diego University of California [44].

Recently, a systematic PubMed search was conducted to identify studies reporting clinical outcomes in patients with cancer who were reviewed by MTB. From an analysis of fourteen studies with a total of 3328 cancer patients, patients receiving recommended MTB therapy had significantly better clinical outcomes than those receiving conventional therapy, despite data quality limited by lack of studies prospective randomized controlled trials [9].

However, with regard to NGS genomic testing, clinicians highlight several barriers, such as financial reimbursement for the drug and the genomic test, lack of treatment guidelines, lack of patients’ knowledge about the test, and, importantly, issues concerning the interpretation of the genomic results [45].

This is where the artificial intelligence technology comes in handy, guaranteeing the construction of platforms on which the NGS and clinical data of cancer patients are crossed, helps the MTB in the interpretation of the data and in the personalized choice of treatment [46].

It is desirable that the work of MTBs is supported by technological systems to support clinical decisions. An example is provided by the Molecular Tumor Board Portal (MTBP), which represents a platform for sharing and automated interpretation of NGS data, integrating clinical data, between the seven cancer centers that make up Cancer Core Europe (CCE). Several commercial software are currently available, but in-house solutions are often used to best meet the specific needs of each center [47].

The concept of precision medicine was announced, for the first time globally, by the US President Barack Obama in 2015. This announcement had an impact on global health policy. In particular, the subsequent approval by the US FDA of NGS tests, such as FoundationOne^®^ CDx, in 2017, has increased the desire to set treatments based on genomic analyses [46].

An ad hoc policy that guarantees the economic sustainability of genomic tests and access to drugs (mostly very expensive and reimbursed for other therapeutic indications) also through clinical studies is necessary for MTBs to become a useful tool for precision oncology governance. It is also important to work for the accreditation of qualified laboratories that perform molecular tests, such as NGS, and to guarantee adequate training for all professionals involved in MTB, also thanks to the help of scientific societies [48].

The implementation of NGS in daily practice requires significant investments also on bioinformatics workflows in order to provide rapid results to clinicians [23].

## 7. MTBs: The State of the Art in Italy

According to the Italian workshop, an MTB is composed of a core team comprising medical oncologists (all subspecialties should be represented), pathologists, molecular biologists, geneticists, clinical pharmacologists, hospital pharmacists, and a noncore team composed of radiation oncologists, surgeons, radiologists, nuclear physicians, organ specialists, research nurses, psychologists, bioinformaticians, bioethicists, clinical epidemiologists, and representatives of patients’ organizations [15].

Each MTB should be led by a coordinator assisted by a secretary, and MTB meetings should be periodic, with a predefined schedule [12].

In Italy, the number of MTBs in each region has to be planned based on population numbers [15].

On 14 December 2021, the Budget Committee of the Chamber approved an amendment to the bill for the implementation of the “National recovery and resilience plan” which urges the regions to promote the establishment of an MTB that collaborates with the centers of molecular testing, regional cancer networks [49], as well as with the Ethics Committees [12].

The regions should use the financial resources already available, without further burdening the public finances [50].

MTB reports should include characteristics of the molecular tests; sample information [12]; driver mutations/copy number/structural variations, including fusion genes; targetable molecular alterations; microsatellite instability; mutational burden of the tumor; changes indicating drug resistance; MTB conclusions/recommendations including potentially available clinical trials.

MTB deals not only with the interpretation of genomic data but also with the choice of the biological samples to be tested (i.e., primary tumor, recurrence/metastasis or blood) and with the molecular technology to be used [12] to analyze nucleic acids (DNA or RNA) extracted from the tumor.

The NGS test can be commercial, such as the US-approved FoundationOne CDX test, Food and Drug Administration, or performed in house. A survey from the Italian Society of Pathology (SIAPeC) NGS Network showed that 80% of the 30 centers included pertain to pathology departments, with the remaining laboratories in functional connection with the pathology departments. The centers have one or more parallel massive sequencing tools, mostly based on two technologies, Thermo Fisher and Illumina, with only a limited number of centers that have adopted the Qiagen Platform, currently being phased out. Eight (80%) of the ten centers equipped with a single platform opted for IonTorrent technology, while the tools in the various centers are mainly Illumina; this may depend on the technical characteristics of the technologies and costs. The diagnostics of the centers is not yet fully structured within defined territorial networks, to which diagnostic–therapeutic pathways and MTBs are not always connected [51].

Whichever laboratory performs the NGS test, local pathology or external provider, specific standards must be followed for performing the test. The International Organization for Standardization (ISO) has recently published the new edition of the ISO 15189 standard—Medical laboratories—Requirements for quality and competence. All medical laboratories will be required to comply with ISO 15189:2022 as of 7 December 2025 [52].

The delivery time for MTB reports, which is the period for generating the output, has been settled within 16 days. The median time of MTB experiences reported in the literature, including both molecular analysis and discussion of MTB, was 38.4 days, with a wide range from 12.4 to 86 days. An average value of 28 days (4 weeks), including time for tumor sequencing and MTB discussion, could represent a good compromise given the time needed to perform the techniques (sample preparation, sequencing/molecular analysis) and the development of the MTB (interpretation, discussion, decision) [7]. However, given the urgency of treating cancer patients, a time of 16 days can be considered more appropriate [53].

Not infrequently, the recommendation of the MTB leads to an indication not foreseen by AIFA. Today the methods of access to the drug, when not indicated according to AIFA, refer to Law 648/96, Law 326/2003 art.48 (5% fund), Law 94/98 (off label use), DM 09/07/2017 (therapeutic use for individual patients) and Class Cnn (Class C drugs) [15].

According to ESMO’s recommendations for the use of NGS, ESMO “recommends using genomics-matched off-label medicines only if they have been developed in a country-level or regional-level access program and decision-making process” [23].

Therefore, this type of claim very often goes unapplied in the absence of a clinical trial or compassionate use program.

However, even when a clinical trial might be right for the patient, geographic, economic or social challenges can be obstacles. For example, clinical trials are usually not evenly distributed across the country, leading to difficulties enrolling cancer patients who do not live close to centers where a specific study is ongoing. According to a 2007 survey, only 37% of cancer patients would be willing to travel to be enrolled in a clinical trial in the United States.

The main challenges in applying the MTB indications highlighted by the authors in most of the studies were: a long-time frame for genomic testing and/or MTB output, which increases the risk of clinical deterioration of patients; accessibility to drugs, even in the context of a clinical trial, lack of clinical trials, geographical accessibility [14].

It is therefore desirable to regulate access to medicines and reimbursement by the national health service. The registration of drugs assigned on the basis of the identified molecular alteration and the clinical results of the treatment (efficacy, toxicity) must be carried out within a national platform managed by AIFA. NGS technology should be included in the National Essential Levels of Care (LEA) [15].

The clinical cases discussed in the MTB should be entered in regional registers in which all the clinical and molecular information are reported, as well as any proposed therapies and the response to them.

The individual regional registers should then interface with a national platform accredited by AIFA to manage any reimbursement of the agnostic drugs used [12].

A reorganization of the Italian National Health System would therefore be necessary to regulate access to molecular tests and drugs with a molecular target and possibly enrolment in clinical trials [54].

Preliminary results of a study of two Italian hospitals on the use of NGS profiling for patients with non-small cell lung cancer showed a full cost per NGS test of €1150, versus a cost of €1780 for standard methods. The full cost for NGS testing, if TMB mapping were also obtained, would be approximately EUR 1850 [15]. It is necessary to identify and accredit the centers for carrying out the NGS tests and to define their inclusion in the LEA at a national level and to assign the DRGs with the relative tariffs at a regional level. The first two regions that have defined tariffs for the execution of NGS tests for diagnostic purposes are Lombardia and Emilia-Romagna. Starting from the experience of these two regions, it is possible to deduce that the fee for the sequencing of many genes is cheaper and more informative than the sum of the fees for the tests focused on single mutations. However, the cost of the test represents, on average, a very low share of the overall cost of the diagnostic-therapeutic path of the cancer patient.

Another fundamental element for the establishment and dissemination of MTBs is their national integration to improve the standardization of procedures.

Therefore, the establishment and activities of MTBs must be supported and made uniform and traceable through a virtual consultation system (VCS); the teleplatform must allow online and teleconferencing connection of the oncological structures belonging to the single MTB within the scheduled meetings for discussion and follow-up of clinical cases. With a view to facilitating the sharing of genomic and clinical data of patients in MTBs, an interesting initiative is the virtual consultation system (VCS), a platform developed by the Oncological Pole of Sapienza, by the Research and Health Foundation (ReS) and from CINECA. This platform would allow online meetings of MTBs and the systematic collection of all clinical, imaging, and genomic profiling data of each patient. This platform is currently being activated by the Pascale Institute of Naples, the National Cancer Institute of Milan, and the San Martino of Genoa, also with the aim of reducing the overall costs of precision medicine [55].

The MTB network must be accredited by AIFA, according to transparent criteria and procedures for the composition, activities, traceability, and data processing; the individual MTBs will have to be implemented by the Regions and by the general management and represent the new management tool of the mutational model in oncological practice.

## 8. Conclusions

The MTBs are groups created with the aim of understanding the complex genetic and molecular results of profiling tests and proposing the most appropriate therapeutic strategy based on the active drugs available. Economic availability for genomic testing, access to drugs or clinical trials according to the MTB recommendations and the extensive use of existing anticancer drugs are necessary for MTB to become a useful tool for the governance of precision oncology. At the same time, scientific, and clinical skills capable of following the complex and rapid evolution of cancer genetics and the availability of new or experimental anticancer therapies must be guaranteed [48].

CIPOMO (Italian Association of Heads of Oncology Department) promoted a national survey to photograph the current state of precision oncology in Italy in which centers from 19 regions out of 21 participated. A total of 33.6% of the participating oncologists did not have access to MTB. NGS and MTB technologies are distributed unevenly in Italy, hindering equal opportunities for patients to access innovative therapies. The construction of a national database collecting the clinical activities of MTBs could objectify their impact on patient pathways and outcomes [56].

As far as MBC is concerned, it can be stated that, to date, it is not necessary to perform NGS multigene analysis on tumor tissue for the choice of the first line of therapy, since the *BRCA1/BRCA2* status can be determined by germline DNA sequencing, PD-L1 status by immunohistochemistry and *PIK3CA* by tumor tissue PCR.

Level II ESCAT alterations that appear to confer clinical benefit are: *ESR1* [57], *AKT1* and *PTEN* alterations [58], and *ERBB2* mutations [59].

However, considering the high number of level II alterations according to the ESCAT scale, it is important to include patients in molecular screening programs that exploit NGS techniques, thus granting them access to studies that test targeted therapies for specific genomic alterations that may have been identified. Literature data demonstrate that the percentage of patients treated on the basis of molecular tests in clinical trials is low (2–18%) [42].

So, said data show that breast cancer is discussed in MTB in 20% of cases [7].

The data demonstrating the benefit of MTBs are scarce, considering that to date no study has been constructed by carrying out a randomization by outcome related to the decision of MTB or not, making it difficult to determine the real efficacy of MTBs. However, MTBs cannot cause damage but at a high cost.

The expenditure of the National Health System for NGS tests is EUR 29.9 million (an estimated 14,421 metastatic patients/year are eligible for the NGS test which costs EUR 2073 as per the reimbursement rate set by the Lombardy Region); to this expense is added the expense for off-label oncological drugs (1531 patients are estimated to be candidates for off-label drugs with an average monthly cost of EUR 3000 for a median duration of 5 months) of EUR 22.3 million, for an overall annual budget of EUR 52.2 million. The costs of the NGS tests are progressively decreasing from EUR 1500 to 500 depending on the method used; and the number of patients is also decreasing since the profiling criteria specify that eligible patients must have an ECOG performance status < 2 and a life expectancy of at least 6 months [55].

In a careful cost/benefit analysis, it must be considered that NGS techniques have a cost and that the results of molecular tests could lead to the recommendation of expensive drugs outside their approved indication [23]. In terms of cancer healthcare costs, breast cancer accounts for approximately 13% in the European Union, the highest of all cancers and the second largest overall economic burden after lung cancer [4]. Furthermore, the costs increase if we include those of molecular tests. This reinforces the importance of discussing individual clinical cases in MTB.

Genomic profiling should only be performed as part of routine clinical practice if the outcome will change the treatment approach, as guided by the ESCAT scale, or if the patient has potential access to clinical trials [5].

Adequately translating and interpreting the complex genetic and molecular characteristics of the neoplasm into information that clinicians can use to propose the most appropriate and individualized treatment is critical not only from a strictly health point of view but also from an economic sustainability point of view for the country.

This profound transformation that is mutational oncology is the logic that guides the implementation of MTB.

In conclusion, MTBs make it possible to set the best oncological therapy for the individual patient to overcome the limits of the interpretation of genomic alterations and the availability of targeted drugs also thanks to access to clinical studies. In studying the individual patient, we must not forget that tumors are enriched over time with genomic alterations. Thus, the primary tumor tissue may not be representative of the genomic profile of advanced disease suggesting the importance of acquiring multiple longitudinal tissue biopsies.

In Italy, the NGS analysis is heterogeneous; therefore, the institutions must set themselves the goal of harmonizing the network of laboratories. Furthermore, the agreement in therapeutic recommendations among MTBs worldwide is not unique because MTBs differ in terms of composition, methods, and opportunities for available therapies [60].

## Figures and Tables

**Table 1:** The level I/II genomic alterations of metastatic breast cancer according to the ESCAT scale.

Genomic Alteration	Level
*ERBB2* amplification	I
Germline *BRCA1/BRCA2* mutations	I
*PIK3CA* mutations	I
Microsatellite instability (MSI)	I
*NTRK* translocations	I
*ESR1* mutations	II
*PTEN* loss	II
*AKT1* mutations	II
*ERBB2* mutations	II

**Table 2:** Molecular screening studies.

Author/Study	Type of Metastatic Tumor (n, %)	Enrolled Patients (n)	Evaluable Patients (n)	Patients with Actionable Alterations (n, %)	Patients Receiving Targetable Therapies (n, %)	Results
Massard et al. [27]/ Moscato-01	solid tumors (breast: 135, 14%)	948	843	411, 49%	199, 27%	PFS2/PFS1 > 1.3. Sixty-three out of 948 pts (7%) benefited in PFS.
Andrè et al. [28]/ Safir01/UNICANCER	breast (423, 100%)	423	297	195, 66%	55, 19%	Four out of 43 patients (9%) had an objective response. Nine (21%) had stable disease for more than 16 weeks.
Pezo et al. [29]	breast (483, 100%)	483	440	203, 46%	15%	No difference in median time on treatment between patients treated with matched therapies and those with unmatched therapies (3.6 vs. 3.8 months, *p* = 0.89).
Pernas et al. [30]/ SOLTI-1301 AGATA	breast (305, 100%)	305	260	123, 47%	13, 5%	Among 13 patients, 46.2% had PFS ≥ 6 months with combination therapy.
Parker et al. [31]	breast (43, 100%)	43	40	17, 42%	17, 42%	PFS was significantly worse for patients receiving a therapy not matched with the identified genomic alteration.
Walter et al. [32]	breast (52, 100%)	52	52	45, 87%	22, 42%	-
Aftimos et al. [33]/ AURORA	breast (381, 100%)	381	88%	51%	7%	-
Le Tourneau et al. [34]/ SHIVA	solid tumor (breast: 59, 20%)	741	741	293, 39.5%	195, 26%	The use of targeted drugs did not statistically significantly improve PFS (HR = 0.88; *p* = 0.41).
Andrè et al. [35]/ SAFIR02-BREAST and SAFIR-PI3K	breast (1462, 100%)	1462	238	115, 48%	-	Median PFS was 9.1 and 2.8 months in the targeted therapy and chemotherapy arms, respectively (HR = 0.41; *p* < 0.001).
Hlevnjak et al. [36]/ CATCH	breast (200, 100%)	200	128	64, 50%	53, 41%	Twenty-one out of 53 patients (40%) achieved stable disease (n = 13.25%) or partial response (n = 8.15%). Sixteen (30%) of those patients showed PFS improvement of at least 30% during MTB-recommended treatment compared to PFS of the previous line of treatment.
Bruzas et al. [37]	breast (95, 100%)	95	83	63, 76%	30, 36%	The ratio of PFS in NGS-based therapy to PFS in the last line of standard therapy before NGS was >1.3 of 13 (43.3%) patients, indicative of a clinical benefit to NGS-directed therapy. The one-year overall survival rates were 22.7% in the 65 patients assigned to standard therapy compared with 62.9% in the 30 patients who received combination therapy.
Fukada et al. [38]	310 (breast: 37, 100%)	37	35	30, 86%	9, 26%	-
Turner et al. [39]/ plasmaMATCH	breast (1051, 100%)	1051	1034	-	Cohort A: 74, 7.2% Cohort B: 20, 1.9% Cohort C: 18, 1.7% Cohort D: 19, 1.8%	Five (25%) of 20 patients in cohort B and four (22%) of 18 patients in cohort C having a response. Six (8%) of 74 in cohort A and two (11%) of 19 patients in cohort D having a response.
van Geelen et al. [40]	breast (322, 100%)	322	234	171, 73%	74, 32%	Patients with a higher number of mutations had significantly worse overall survival.
Botticelli et al. [24]/ ROME	solid tumor (breast: 62, 6.3%)	62	62	34, 55%	28, 45%	Germline mutations have been identified in patients with no prior indication for germline testing.

PFS: progression-free survival; PFS2: progression-free survival on therapy decided by MTB; PFS1: progression-free survival on therapy before that decided by MTB; MTB: molecular tumor board; NGS: next-generation sequencing.

## References

[1] Di Pilla A., Cozzolino M.R., Mannocci A., Carini E., Spina F., Castrini F., Grieco A., Messina R., Damiani G., Specchia M.L. (2022). The Impact of Tumor Boards on Breast Cancer Care: Evidence from a Systematic Literature Review and Meta-Analysis. Int. J. Environ. Res. Public Health.

[2] Gori S., Miglietta F., Modena A. (2021). Mammella. I numeri del cancro in Italia.

[3] Gori S., Miglietta F., Biganzoli L., Calabrese M., Cortesi L., Conte B., Criscitiello C., Del Mastro L., Dieci M.V., Folli S. (2021). Linee guida “Neoplasie della mammella”.

[4] Biganzoli L., Cardoso F., Beishon M., Cameron D., Cataliotti L., Coles C.E., Delgado Bolton R.C., Die Trill M., Erdem S., Fjell M. (2020). The requirements of a specialist breast centre. Breast.

[5] Gennari A., André F., Barrios C.H., Cortés J., de Azambuja E., De Michele A., Dent R., Fenlon D., Gligorov J., Hurvitz S.A. (2021). ESMO Clinical Practice Guideline for the diagnosis, staging and treatment of patients with metastatic breast cancer. Ann. Oncol..

[6] Gradishar W.J., Moran M.S., Abraham J., Abramson V., Aft R., Agenese D., Allison K.H., Andreson B., Burstein H.J., Chew H. (2023). NCCN Clinical Practice Guidelines in Oncology (NCCN Guidelines^®^). Breast Cancer. Version 2.2023. www.nccn.org/patients.

[7] Luchini C., Lawlor R.T., Milella M., Aldo Scarpa A. (2020). Molecular Tumor Boards in Clinical Practice. Trends Cancer..

[8] Frost H., Graham D.M., Carter L., O’Regan P., Landers D., Freitas A. (2022). Patient attrition in Molecular Tumour Boards: A systematic review. Br. J. Cancer.

[9] Larson K.L., Huang B., Weiss H.L., Hull P., Westgate P.M., Miller R.W., Arnold S.M., Kolesar J.M. (2021). Clinical Outcomes of Molecular Tumor Boards: A Systematic Review. JCO Precis. Oncol..

[10] Martini N., Piccinni C. (2021). The tools for the governance of mutational oncology and agnostic drugs: From place in therapy to place in pathway. Recenti Prog Med..

[11] Sultova E., Westphalen C.B., Jung A., Kumbrink J., Kirchner T., Mayr D., Rudelius M., Ormanns S., Heinemann V., Metzeler K.H. (2021). Implementation of Precision Oncology for Patients with Metastatic Breast Cancer in an Interdisciplinary MTB Setting. Diagnostics.

[12] Russo A., Incorvaia L., Beretta G., Chiari R., Cinieri S., Ferrara R., Galvano A., Gori S., Guadagni F., Marchetti P. (2020). Raccomandazioni AIOM “Tumor Board Molecolare”.

[13] Schwartzberg L., Kim E.S., Liu D., Schrag D. (2017). Precision Oncology: Who, How, What, When, and When Not?. Am. Soc. Clin. Oncol. Educ. Book.

[14] Crimini E., Repetto M., Tarantino P., Ascione L., Antonarelli G., Guerini Rocco E., Barberis M., Mazzarella L., Curigliano G. (2022). Challenges and Obstacles in Applying Therapeutical Indications Formulated in Molecular Tumor Boards. Cancers.

[15] Pinto C., Biffoni M., Popoli P., Marchetti A., Marchetti P., Martini N., Normanno N. (2021). Molecular tests and target therapies in oncology: Recommendations from the Italian workshop. Future Oncol..

[16] Andre F., Mardis E., Salm M., Soria J.C., Siu L.L., Swanton C. (2014). Prioritizing targets for precision cancer medicine. Ann Oncol..

[17] Van Allen E.M., Wagle N., Stojanov P., Perrin D.L., Cibulskis K., Marlow S., Jane-Valbuena J., Friedrich D.C., Kryukov G., Carter S.L. (2014). Whole-exome sequencing and clinical interpretation of formalin-fixed, paraffin-embedded tumor samples to guide precision cancer medicine. Nat. Med..

[18] Dienstmann R., Sock Jang I., Bot B., Friend S., Guinney J. (2015). Database of genomic biomarkers for cancer drugs and clinical targetability in solid tumors. Cancer Discov..

[19] Sukhai M.A., Craddock K.J., Thomas M., Hansen A.R., Zhang T., Siu L., Bedard P., Stockley T.L., Kamel-Reid S. (2016). A classification system for clinical relevance of somatic variants identified in molecular profiling of cancer. Genet. Med..

[20] Chakravarty D., Gao J., Phillips S.M., Kundra R., Zhang H., Wang J., Rudolph J.E., Yaeger R., Soumerai T., Nissan M.H. (2017). OncoKB: A Precision Oncology Knowledge Base. JCO Precis. Oncol..

[21] Li M.M., Datto M., Duncavage E.J., Kulkarni S., Lindeman N.I., Roy S., Tsimberidou A.M., Vnencak-Jones C.L., Wolff D.J., Younes A. (2017). Standards and Guidelines for the Interpretation and Reporting of Sequence Variants in Cancer: A Joint Consensus Recommendation of the Association for Molecular Pathology, American Society of Clinical Oncology, and College of American Pathologists. J. Mol. Diagn..

[22] Condorelli R., Mosele F., Verret B., Bachelot T., Bedard P.L., Cortes J., Hyman D.M., Juric D., Krop I., Bieche I. (2019). Genomic alterations in breast cancer: Level of evidence for actionability according to ESMO Scale for Clinical Actionability of molecular Targets (ESCAT). Ann. Oncol..

[23] Mosele F., Remon J., Mateo J., Westphalen C.B., Barlesi F., Lolkema M.P., Normanno N., Scarpa A., Robson M., Meric-Bernstam F. (2020). Recommendations for the use of next-generation sequencing (NGS) for patients with metastatic cancers: A report from the ESMO Precision Medicine Working Group. Ann. Oncol..

[24] Botticelli A., Scagnoli S., Conte P., Cremolini C., Ascierto P.A., Cappuzzo F., Aglietta M., Mazzuca F., Capoluongo E., Blandino G. (2023). Mutational landscape of breast cancer patients in ROME trial: Preliminary results. Cancer Res..

[25] Trotta F., Traversa G. (2016). La Ricerca di Precisione tra Ombrelli e Cestini. https://forward.recentiprogressi.it/wp-content/uploads/2016/08/recprogrmed_2016_suppl1_trotta_traversa.pdf.

[26] Lewis C., Harvey R.D. (2020). Precision Oncology Comes of Age: Tumor-Agnostic Approaches. J. Adv. Pract. Oncol..

[27] Massard C., Michiels S., Ferté C., Le Deley M.C., Lacroix L., Hollebecque A., Verlingue L., Ileana E., Rosellini S., Ammari S. (2017). High-Throughput Genomics and Clinical Outcome in Hard-to-Treat Advanced Cancers: Results of the MOSCATO 01 Trial. Cancer Discov..

[28] André F., Bachelot T., Commo F., Campone M., Arnedos M., Dieras V., Lacroix-Triki M., Lacroix L., Cohen P., Gentien D. (2014). Comparative genomic hybridisation array and DNA sequencing to direct treatment of metastatic breast cancer: A multicentre, prospective trial (SAFIR01/UNICANCER). Lancet Oncol..

[29] Pezo R.C., Chen T.W., Berman H.K., Mulligan A.M., Razak A.A., Siu L.L., Cescon D.W., Amir E., Elser C., Warr D.G. (2018). Impact of multi-gene mutational profiling on clinical trial outcomes in metastatic breast cancer. Breast Cancer Res. Treat..

[30] Pernas S., Villagrasa P., Vivancos A., Scaltriti M., Rodón J., Burgués O., Nuciforo P., Canes J., Paré L., Dueñas M. (2021). First Nationwide Molecular Screening Program in Spain for Patients with Advanced Breast Cancer: Results from the AGATA SOLTI-1301 Study. Front. Oncol..

[31] Parker B.A., Schwaederlé M., Scur M.D., Boles S.G., Helsten T., Subramanian R., Schwab R.B., Kurzrock R. (2015). Breast Cancer Experience of the Molecular Tumor Board at the University of California, San Diego Moores Cancer Center. J. Oncol. Pract..

[32] Walter C., Hartkopf A., Koch A., Klaumünzer M., Schulze M., Grischke E.M., Taran F.A., Brucker S., Battke F., Biskup S. (2020). Sequencing for an interdisciplinary molecular tumor board in patients with advanced breast cancer: Experiences from a case series. Oncotarget.

[33] Aftimos P., Oliveira M., Irrthum A., Fumagalli D., Sotiriou C., Nili Gal-Yam E., Robson M.E., Ndozeng J., Di Leo A., Ciruelos E.M. (2021). Genomic and Transcriptomic Analyses of Breast Cancer Primaries and Matched Metastases in AURORA, the Breast International Group (BIG) Molecular Screening Initiative. Cancer Discov..

[34] Le Tourneau C., Delord J.P., Gonçalves A., Gavoille C., Dubot C., Isambert N., Campone M., Trédan O., Massiani M.A., Mauborgne C. (2015). Molecularly targeted therapy based on tumour molecular profiling versus conventional therapy for advanced cancer (SHIVA): A multicentre, open-label, proof-of-concept, randomised, controlled phase 2 trial. Lancet Oncol..

[35] André F., Gonçalves A., Filleron T., Dalenc F., Lusque A., Campone M., Sablin M.P., Bonnefoi H., Bieche I., Lacroix L. (2022). Clinical utility of molecular tumor profiling: Results from the randomized trial SAFIR02-BREAST. Cancer Res..

[36] Hlevnjak M., Schulze M., Elgaafary S., Fremd C., Michel L., Beck K., Pfütze K., Richter D., Wolf S., Horak P. (2021). CATCH: A Prospective Precision Oncology Trial in Metastatic Breast Cancer. JCO Precis. Oncol..

[37] Bruzas S., Kuemmel S., Harrach H., Breit E., Ataseven B., Traut A., Rüland A., Kostara A., Chiari O., Dittmer-Grabowski C. (2021). Next-Generation Sequencing-Directed Therapy in Patients with Metastatic Breast Cancer in Routine Clinical Practice. Cancers.

[38] Fukada I., Mori S., Hayashi N., Hosonaga M., Yamazaki M., Wang X., Kawai S., Inagaki L., Ozaki Y., Kobayashi K. (2022). Assessment of a cancer genomic profile test for patients with metastatic breast cancer. Sci. Rep..

[39] Turner N.C., Kingston B., Kilburn L.S., Kernaghan S., Wardley A.M., Macpherson I.R., Baird R.D., Roylance R., Stephens P., Oikonomidou O. (2020). Circulating tumour DNA analysis to direct therapy in advanced breast cancer (plasmaMATCH): A multicentre, multicohort, phase 2a, platform trial. Lancet Oncol..

[40] van Geelen C.T., Savas P., Ling Teo Z., Luen S.J., Weng C.F., Ko Y.A., Kuykhoven K.S., Caramia F., Salgado R., Francis P.A. (2020). Clinical implications of prospective genomic profiling of metastatic breast cancer patients. Breast Cancer Res..

[41] The Rome Trial from Histology to Target: The Road to Personalize Target Therapy and Immunotherapy (ROME). https://clinicaltrials.gov/ct2/show/NCT04591431.

[42] Kurnit K.C., Dumbrava E.E.I., Litzenburger B., Khotskaya Y.B., Johnson A.M., Yap T.A., Rodon J., Zeng J., Shufean M.A., Bailey A.M. (2018). Precision Oncology Decision Support: Current Approaches and Strategies for the Future. Clin. Cancer Res..

[43] Angel M.O., Pupareli C., Soule T., Tsou F., Leiva M., Losco F., Esteso F., O Connor J.M., Luca R., Petracci F. (2021). Implementation of a molecular tumour board in LATAM: The impact on treatment decisions for patients evaluated at Instituto Alexander Fleming, Argentina. Ecancermedicalscience.

[44] Kato S., Kim K.H., Lim H.J., Boichard A., Nikanjam M., Weihe E., Kuo D.J., Eskander R.N., Goodman A., Galanina N. (2020). Real-world data from a molecular tumor board demonstrates improved outcomes with a precision N-of-One strategy. Nat. Commun..

[45] Barroso-Sousa R., Guo H., Srivastava P., James T., Birch W., Siu L.L., Tew W.P., Tolaney S.M. (2019). Utilization of tumor genomics in clinical practice: An international survey among ASCO members. Future Oncol..

[46] Hamamoto R., Koyama T., Kouno N., Yasuda T., Yui S., Sudo K., Hirata M., Sunami K., Kubo T., Takasawa K. (2022). Introducing AI to the molecular tumor board: One direction toward the establishment of precision medicine using large-scale cancer clinical and biological information. Exp. Hematol. Oncol..

[47] Tamborero D., Dienstmann R., Rachid M.H., Boekel J., Lopez-Fernandez A., Jonsson M., Razzak A., Braña I., De Petris L., Yachnin J. (2022). The Molecular Tumor Board Portal supports clinical deci-sions and automated reporting for precision oncology. Nat. Cancer.

[48] Incorvaia L., Russo A., Cinieri S. (2022). The molecular tumor board: A tool for the governance of precision oncology in the real world. Tumori.

[49] Gramolini E. (2021). Oncologia di Precisione, sì ai Molecular Tumor Board. Panorama della Sanità.

[50] (2021). Bollettino Delle Giunte E Delle Commissioni Parlamentari. http://documenti.camera.it/leg18/resoconti/commissioni/bollettini/pdf/2021/12/14/leg.18.bol0713.data20211214.pdf.

[51] Marchetti A., Barbareschi M., Barberis M., Buglioni S., Buttitta F., Fassan M., Fontanini G., Marchiò C., Papotti M., Pruneri G. (2021). Real-World Data on NGS Diagnos-tics: A survey from the Italian Society of Pathology (SIAPeC) NGS Network. Pathologica.

[52] (2022). Medical Laboratories—Requirements for Quality and Competence.

[53] Wolff L., Kiesewetter B. (2022). Applicability of ESMO-MCBS and ESCAT for Molecular Tumor Boards. memo.

[54] Curigliano G., Ciliberto G., Criscitiello C., Lazzari C., Lorusso D., Montemurro F., Nanni O., Tommasi S. (2020). Capitolo 7: Accesso ai Farmaci. Linee Guida per l’istituzione e la gestione dei Molecular Tumor Board negli Istituti di Alleanza Contro il Cancro. https://www.alleanzacontroilcancro.it/wp-content/uploads/2021/03/Linee-guida.pdf.

[55] Martini N., De Maria R., Amunni G., Apolone G., Beretta G., Bertetto O., Blasi L., Ciliberto G., Cinieri S., Conte P.F. (2020). Documento di consenso sullo sviluppo e sull’organizzazione dell’oncologia mutazionale in Italia. Il Pensiero Scientifico Editore. Suppl. A Politiche Sanit..

[56] Fasola G., Barducci M.C., Pelizzari G., Grossi F., Pinto C., Daniele B., Giordano M., Ortega C., Silva R.R., Tozzi V.D. (2023). Implementation of Precision Oncology in Clinical Practice: Results of a National Survey for Health Care Professionals. Oncologist.

[57] Bardia A., Bidard F.C., Neven P., Streich G., Montero A.J., Forget F., Mouret-Reynier M.A., Sohn J.H., Taylor D., Harnden K.K. (2023). EMERALD phase 3 trial of elacestrant versus standard of care endo-crine therapy in patients with ER+/HER2- metastatic breast cancer: Updated results by duration of prior CDK4/6i in metastatic setting. Cancer Res..

[58] Turner N.C., Oliveira M., Howell S., Dalenc F., Cortes J., Gomez H., Hu X., Jhaveri K., Loibl S., Morales Murillo S. (2023). Capivasertib and fulvestrant for patients with aromatase inhibitor-resistant hormone receptor-positive/human epi-dermal growth factor receptor 2-negative advanced breast cancer: Results from the Phase III CAPItello-291 trial. Cancer Res..

[59] Saura C., Oliveira M., Feng Y.-H., Dai M.S., Chen S.W., Hurvitz S.A., Kim S.B., Moy B., Delaloge S., Gradishar W. (2020). Neratinib Plus Capecitabine Versus Lapatinib Plus Capecitabine in HER2-Positive Metastatic Breast Cancer Previously Treated with ≥ 2 HER2-Directed Regimens: Phase III NALA Trial. J. Clin. Oncol..

[60] Falcone R., Lombardi P., Filetti M., Fabi A., Altamura V., Scambia G., Daniele G. (2023). Molecular Profile and Matched Targeted Therapy for Advanced Breast Cancer Patients. Curr. Oncol..

